# Multispectral imaging for characterizing autofluorescent tissues

**DOI:** 10.1038/s41598-024-61020-7

**Published:** 2024-05-27

**Authors:** Sara Bentahar, María Victoria Gómez-Gaviro, Manuel Desco, Jorge Ripoll, Roberto Fernández

**Affiliations:** 1 Departamento de Bioingeniería, Universidad Carlos III de Madrid, Madrid, Spain; 2 Instituto de Investigación Sanitaria Gregorio Marañón, Madrid, Spain; 3 Centro de Investigación Biomédica en Red de Salud Mental (CIBERSAM), Madrid, Spain; 4 Centro Nacional de Investigaciones Cardiovasculares Carlos III (CNIC), Madrid, Spain; 5 Departamento de Física, Ingeniería de Sistemas y Teoría de la Señal, Universidad de Alicante, Alicante, Spain

**Keywords:** Light-sheet microscopy, Optical techniques, Imaging and sensing, Anatomy

## Abstract

Selective Plane Illumination Microscopy (SPIM) has become an emerging technology since its first application for 3D in-vivo imaging of the development of a living organism. An extensive number of works have been published, improving both the speed of acquisition and the resolution of the systems. Furthermore, multispectral imaging allows the effective separation of overlapping signals associated with different fluorophores from the spectrum over the whole field-of-view of the analyzed sample. To eliminate the need of using fluorescent dyes, this technique can also be applied to autofluorescence imaging. However, the effective separation of the overlapped spectra in autofluorescence imaging necessitates the use of mathematical tools. In this work, we explore the application of a method based on Principal Component Analysis (PCA) that enables tissue characterization upon spectral autofluorescence data without the use of fluorophores. Thus, enabling the separation of different tissue types in fixed and living samples with no need of staining techniques. Two procedures are described for acquiring spectral data, including a single excitation based method and a multi-excitation scanning approach. In both cases, we demonstrate the effective separation of various tissue types based on their unique autofluorescence spectra.

## Introduction

Selective Plane Illumination Microscopy (SPIM) is a fluorescence imaging technique with increasing applications in the biomedical field as it provides high depth resolution with minimal phototoxicity^1–4^. Its magnificent resolution at high penetration depth and the fast acquisition rate, causing minimal disruption to the sample, allows the monitoring of living samples and their biological dynamic processes^4–6^. By acquiring information on fluorescence emission for every pixel in a focal plane simultaneously, it allows 3D visualization of transparent specimens due to the acquisition of z-stacks which can be analyzed and reconstructed into a 3D volume. Since this is a fluorescence imaging technique, it relies mainly on the detection of the fluorescence signal emitted by fluorophores labelling specific structures or tissues. Thus, many studies use a combination of several fluorescent markers to obtain multi-color images, which can provide information on cellular interactions^6,7^. In these cases, where there is more than one fluorophore present in the same sample, overlapping of the fluorophores spectra is unavoidable and requires the use of different techniques, such as linear unmixing^8,9^, to separate the components of the image. Although this technique is commonly used in fluorescence microscopy, it is based on the previous knowledge of fluorescence behavior to separate and group the pixels conforming multispectral images^8,10^. In this sense, multispectral, or hyperespectral, imaging has proven to be a powerful tool to separate overlapped spectra by acquiring spatial and spectral information from the sample. Its major applications include diagnostic imaging and multi color imaging with multiple fluorescent markers^9,11,12^. However, in some of these methods, the light needs to be focused onto a slit or dispersion grating. Thus, to generate each 2D image, it is needed to scan the whole sample in a time-consuming process by acquiring the line within the detection plane^9,12^. Moreover, the use of fluorophores in these techniques to label the structures to be detected, despite being an effective procedure for visualizing biological processes, is an expensive technique that may induce alterations in the natural metabolism of the organisms depending on the concentration of the dyes used^13,14^.

On the other hand, multispectral imaging can be combined with autofluorescence imaging to eliminate the necessity of using a fluorescent dye for visualization^15–17^. The data collected through this modality can be analyzed with mathematical tools to separate the sources emitting every specific emission response by grouping pixels with similar contributions to the transmitted light.

In this work, we develop a method to analyze multispectral images acquired with a SPIM system, aiming to distinguish different tissue types in both fixed and in-vivo samples, without the reliance on fluorescent markers. Instead, we utilize the inherent autofluorescence emission of each tissue as the variable for their characterization. As there are no fluorescent markers in the samples under study, the mathematical tool used for spectral unmixing is Principal Component Analysis (PCA) as it does not need previous spectra knowledge to determine the principal components (PCs)^18–20^. The extracted PCs can be directly related to specific tissues or structures. Combining PCA with multispectral imaging techniques, provides necessary tools for label-free tissue analysis, enabling non-invasive and comprehensive investigations of biological systems and pathological conditions.

## Materials and methods

### Experimental setup

**Figure 1:**
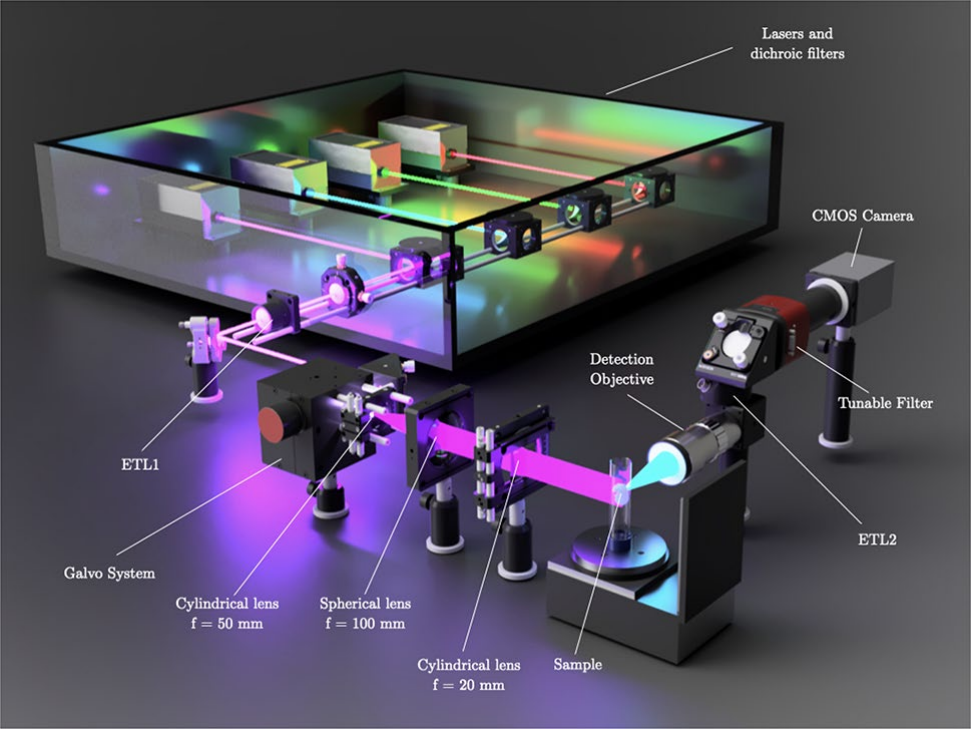
Custom-made multispectral Light sheet microscopy system architecture and components.

A custom-made multispectral light sheet microscopy system was used^21–23^ (Fig. 1). This setup had four different excitation laser lines (402 nm, 490 nm, 532 nm, 632 nm) with powers ranging from 40 to 200 mW . The light coming from either one of the four lasers was directed through several kinematic mirrors to a system of two galvanometer mirrors connected to a computer, allowing precise control of the position of the images on the camera sensor.

To create the light sheet, a combination of lenses, which consisted of two cylindrical lenses (f = 50.0 mm and f = 20.0 mm, respectively) and a spherical lens (f = 100.0 mm) in between them, was used. The first cylindrical lens generates the plane of light from the incoming beam, then the spherical lens is used to focus the laser under the objective and the second cylindrical lens redirects the light sheet onto the focal spot. Autofluorescence emitted by the specimen was captured by the detection objective, which was perpendicular to the incident light. An Optotune EL-16-40 electrically tunable lens (ETL1) was used to ensure optimum light focus on a single spot of the camera by adjusting the focal length, compensating for the axial focal shift caused by the optical path differences after passing through the sample. Before reaching the camera (Hamamatsu ORCA-Flash4.0), a Kurios-WB1 Liquid Crystal tunable filter (Thorlabs) was placed between the objective and the camera in the detection axis. Thus, allowing multispectral imaging by precisely controlling the wavelength which is filtered before reaching the CMOS sensor of the camera.

Samples were positioned by means of three motorized translation stages (X, Y, and Z axes) and a rotation motor (Zaber Technologies). The inclusion of the previously mentioned galvo motors enabled 3D scanning of the sample with a static sample stage, once the sample was positioned. The first galvo motor scanned the sample in the vertical direction, while in combination with ETL2 in the detection arm, the laser plane could scan the entire sample without the need for vertical displacement.

For controlling the whole system and acquiring the image, a software provided by Planelight S.L was used in combination with a DAQ device (Native Instruments). Thus, through this software it was possible to select the excitation wavelength, the voltage sent to the electrically tunable lenses to change their focus, or controlling the galvo motors and linear stages. In addition, a first post-processing was also performed through this software in order to reconstruct the 3D images from the slices acquired from the sample.

It is worth noting that a preprocessing and calibration procedure was applied to the acquired images. Due to the wavelength-dependent properties of filter transmission and camera quantum efficiency, we modeled the system response based on information provided by the camera and tunable filter manufacturers. This model was subsequently applied during the preprocessing stage, where each acquired image was divided by the corresponding system response. This operation was performed depending on the filtered emission wavelength to to achieve a continuous system response for the desired wavelengths. Additionally, potential background noise was reduced by removing the background from the images.

### Multispectral imaging

In this work, three different analyses, based on the excitation and characteristics of the sample, were studied. First, we validated the algorithm for tissue segmentation by using a fixed mouse whole-body sample and single-excitation. Then, we compared the single-excitation with multi-excitation acquisition for this sample. Finally, the algorithm was tested for in-vivo imaging and 3D imaging of the *Neocaridina davidi* shrimp^24,25^. This small shrimp species has received limited research attention, but its main features, such as its small size, social behavior, and transparent nature, make it an interesting subject for its development analysis in the non-invasive in-vivo research.

Image acquisition was based on sequentially spectral scanning the sample using the tunable filter positioned on the detection axis (Fig. 1). Despite the filter’s capability for dynamic control, in this method validation, wavelength selection was performed manually. The exposure time for a single plane of 1024 $\times$ 1024 of the mouse body was set at 1 s, while each optical section in the shrimp volume was acquired with an exposure time of 0.2 s. Considering the maximum time required by the tunable filter to switch between wavelengths (40 ms), the acquisition time for each spectral band was determined by the camera exposure time in each case. The use of a tunable filter enabled the recording of three-dimensional subsets (*x*, *y*, $\lambda$), containing spatial information of the whole sample at every excitation wavelength under study^26^. Spatial information was represented by 2D images acquired from the sample at different emission wavelengths. Therefore, spectral data represents the third dimension in the data cube generated. Throughout all conducted experiments, the spectral bands were evenly spaced with a 5 nm difference.

Moreover, to ensure that the light captured by the CMOS camera specifically represented autofluorescence emission, a 20 nm gap was maintained between the excitation laser wavelength and the first scanned wavelength. Consequently, the number of acquired images varied depending on the chosen excitation laser. In all the cases, the final recorded emission wavelength was 730 nm, determined by the characteristics of the tunable filter used. Table 1 contains the range of filtered emission wavelengths for each excitation laser used.

**Table 1:** Excitation lasers and range of scanned wavelengths for each case.

Laser wavelength (nm)	Initial emission wavelength (nm)	Range of scanned wavelengths (nm)
405	425	425–730
488	508	510–730
532	552	550–730
632	652	650–730

After acquisition, the images were processed to characterize the tissue composition in the different samples. As previously stated, considering the abscence of fluorescent markers in the samples, PCA analysis was used for performing spectral unmixing and, therefore, the segmentation and detection of the tissues without prior knowledge of the spectrum.

The analysis utilized the Matlab PCA tool, which employs Eigenvalue Decomposition (EDV) of the covariance matrix. PCA generates a new set of orthogonal axes, projecting pixels with similar values onto these axes. Similarity in pixel values is determined by the tissue’s response to the excitation wavelength, allowing for the association of each tissue with a PC. However, in this study, the extracted variables ranged from 62 to 161, exceeding the actual number of tissues present in any of the analyzed samples. Nonetheless, the first PCs, representing the highest variance, can be related to specific tissues in each sample.

#### Single excitation multispectral imaging

**Figure 2:**
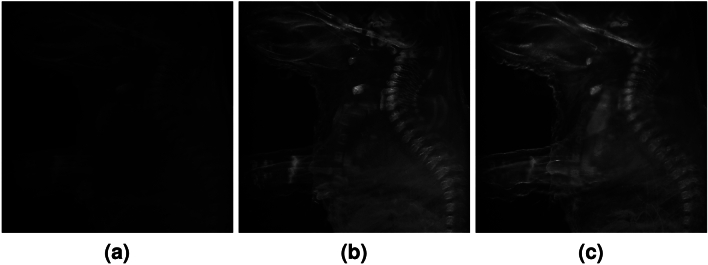
Montage of 3 out of 62 images acquired at 480 nm (**a**), 535 nm (**b**) and 710 nm (**c**) for the single excitation multispectral imaging of the whole mouse embryo.

**Figure 3:**
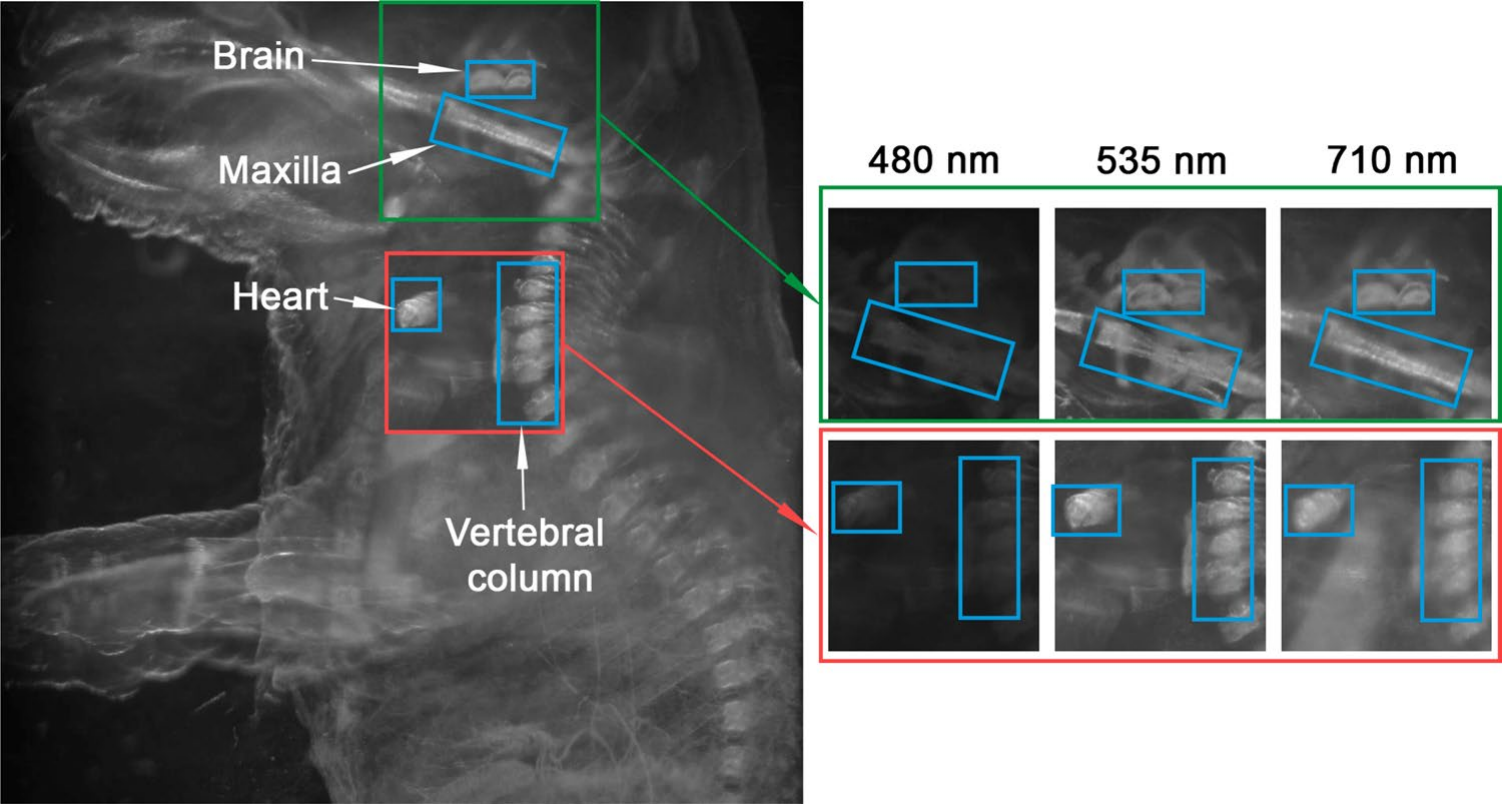
Detail of different areas of the whole mouse embryo acquired at 480 nm, 535 and 710 nm.

For single excitation multispectral imaging, a whole fixed E14.5 mouse embryo of 3 cm was analyzed under an excitation wavelength of 405 nm (Near Ultra Violet, NUV). A total of 62 images, one for each spectral band from 425 to 730 nm in steps of 5 nm, were acquired, representing the spectral behaviour of the different tissues of the sample under NUV illumination. Figure 2 represents a subset of 3 complete images, corresponding to the 480 nm, 535 nm and 710 nm filtered emissions and Fig. 3 the detail of brain, maxilla, heart and vertebral column under these filtered emissions. The figures illustrate the distinct behavior of tissues under the same excitation light. The variation in the filtered emission wavelengths is correlated to a difference in intensity depending on the characteristics of the tissue.

#### Multi-excitation multispectral imaging

In contrast to the single excitation multispectral imaging method previously described, this approach involved the use of multiple lasers to excite the sample at different time points sequentially. This allowed to increase the amount of data available per sample, as additional data cubes were obtained. The data cubes contained spectral bands of different emission wavelengths when excited at a specific wavelength. Thus, each data cube represented the behavior of tissue in response to the incident excitation light. Due to the utilization of a SPIM system equipped with four lasers, a total of four data cubes were obtained

In this multi-excitation approach, we analyzed the whole mouse embryo previously used in the single excitation method to conduct a performance comparison between the two techniques. The sample was excited with the wavelengths detailed in Table 1, acquiring a total of 161 images through this modality. It can be observed the different behaviour of tissues depending not only on the filtered emission wavelength but also on the excitation wavelength. At a 570 nm filtered emission wavelength, the intensity of vertebrae emission is higher when excited with 405 nm than when using 532 nm laser. Similarly, at 720 nm filtered emissions, soft tissue such as kidneys becomes evident when excited using the 632 nm laser, while it remains inappreciable when excited with the other three wavelengths. This had a direct impact on the PCA analysis, particularly in determining the axes of maximum variance, increasing the sensitivity of the system.

**Figure 4:**
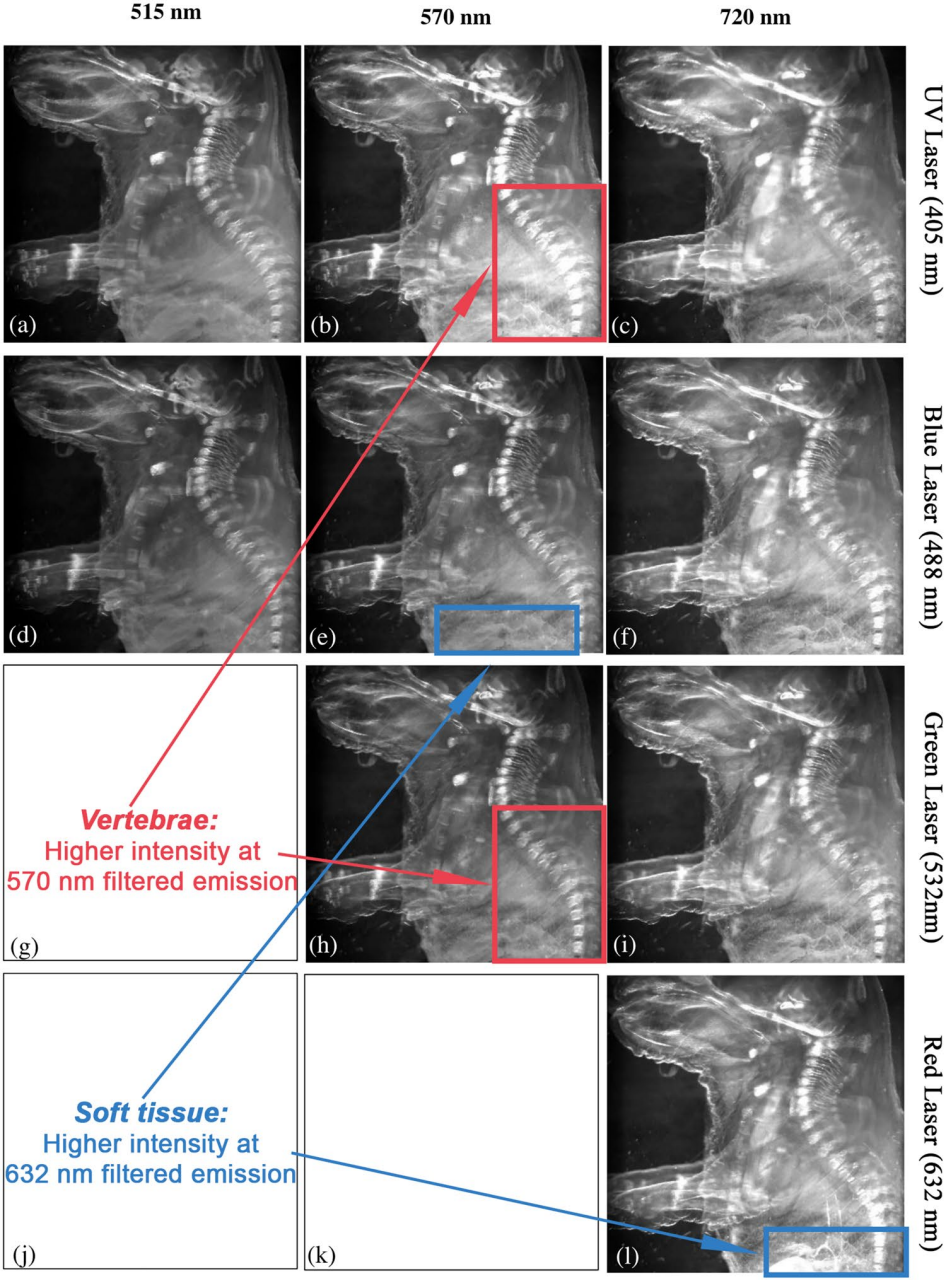
Montage of images of the fixed whole mouse embryo. Images filtered at the same emission wavelength (515, 570 and 720 nm) are shown for every excitation wavelength: 405 nm (**a**–**c**), 488 nm (**d**–**f**), 532 nm (**h**–**i**) and 632 nm (**l**).

Figure 4 represents an example of the images obtained for each excitation wavelength, showcasing the corresponding filtered emission wavelengths.

#### Single-excitation multispectral imaging with 3D reconstruction

After system validation, in-vivo imaging of a live *Neocaridina Davidi* sample was carried out. To minimize the experiment duration, only the single-excitation modality was used, reducing the acquisition time and the number of captured images. Thus, to prevent any possible adverse effects on the organisms, only the 488 nm laser with a maximum power of 2 $\mu$W, was used. Prior to the realization of the experiments, the specimens were sedated. An eugenol solution was used to induce anesthesia on the living shrimps. Eugenol, a sedative derived from plants, is commonly utilized in aquaculture to alleviate psychological stress and minimize potential harm to various species of fish and shellfish during handling, transportation, and research activities^27^.

**Figure 5:**
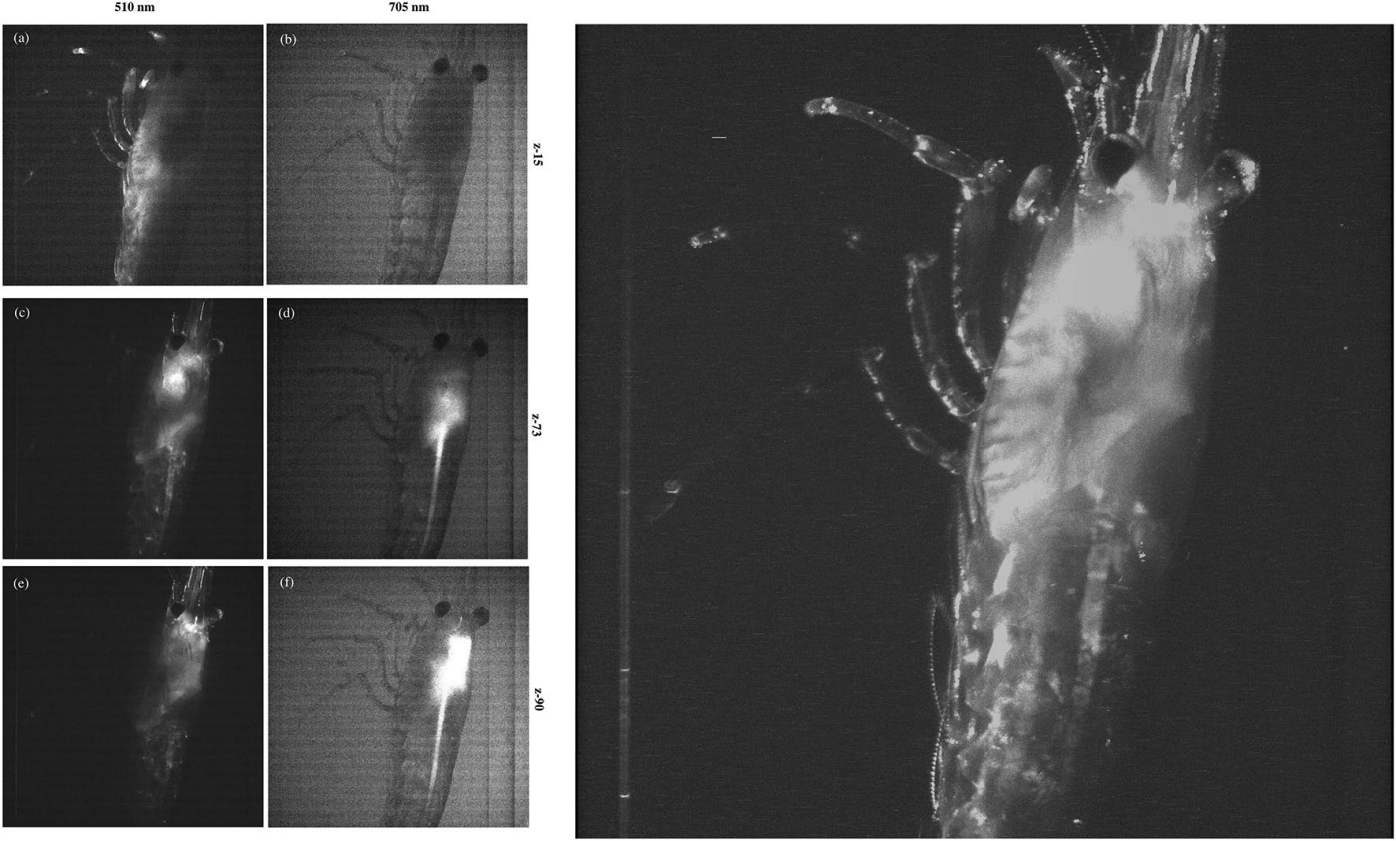
Live shrimp images acquired with a 488 nm laser. Three representative images of z-stacks (z-15, z-73, and z-90) filtered at 510 nm (**a**, **c** and **e**) and 705 nm (**b**, **d** and **f**) (**left**) and 3D reconstruction at 510 nm filtered emission (**right**).

The distinctive factor added in this analysis, besides imaging live samples, is the acquisition of z-stack images, enabling the reconstruction of 3D volume image of the whole specimen. This was accomplished by sequentially displacing the laser beam across the sample, resulting in the generation of optical sections that were subsequently compiled into a stack of images. Specifically, 100 optical sections of 0.0446 mm in depth were taken for every filtered emission wavelength (in the range from 510 nm to 730 nm), producing a total of 4500 images. For each group of 100 optical sections, 3D reconstruction was computed. Figure 5 shows three different optical sections, or z-stacks, for two different filtered emissions, 510 nm and 705 nm and the volume reconstruction of the shrimp at 510 nm filtered emission, using the z-stacks for this specific emission . In (a), the lateral area of the shrimp exhibits a prominent fluorescence signal, while in (b) it shows relatively uniform emission throughout the shrimp tissue. In (e) the head of the shrimp shows the highest fluorescence activity, while for a filtered wavelength of 705 nm, the mid-gut of the shrimp exhibits a concentrated fluorescent signal. These variations underscore the diverse behaviors of shrimp tissues in response to specific emission filtered wavelengths.

#### Principal component analysis of the data

Once the images were acquired, the obtained data cubes were combined into a single matrix to be used used as the input for the PCA analysis tool. These matrices contained all the filtered emissions for each excitation wavelength. The algorithm used for this analysis was based in Eigenvalue Decomposition (EDV) of the covariance matrix. This approach was selected because the number of observations, i.e., the number of pixels, was larger than the number of variables, given by the emission spectral bands.

The analysis produced a new set of orthogonal axes in which pixels with similar values were projected. The similarity between pixel values relies on comparable response of tissue to an excitation wavelength. It is important to note that PCA assigns as many PC as variables and none of the samples analyzed comprised such an extensive range of tissues. Therefore, only the first principal components (representing the maximum variance) were considered since these are the ones related to specific tissues in each sample.

The PC score matrices were obtained as:

1 $$\begin{aligned} P = D \cdot V \end{aligned}$$

where *P* represents the PC scores, *D* is the input matrix and *V* is the matrix of eigenvectors (coefficient matrix).

To reconstruct the original pixel values into the PCs’ axes, it is necessary to multiply the score matrix by the coefficient matrix. Therefore, the centered reconstructed matrix, $Y_{centered}'$ is given by:

2 $$\begin{aligned} coeff \cdot score = Y_{centered}' \end{aligned}$$

$Y_{centered}'$ matrix is a centered matrix, as, by default, the PCA tool centers the input data matrix *X* by subtracting the estimated mean of each variable. To obtain the proper reconstruction, $Y'$, of the original image, the computed mean ($\mu$) was added:

3 $$\begin{aligned} coeff \cdot score + \mu = Y' \end{aligned}$$

$Y'$ matrix is the reconstructed matrix representing the original data replotted in the PCs space, where the axes represent the maximum variance. For multi-excitation images, the reconstructed matrix, $Y'$, was reshaped into a three-dimensional data cube containing four sub-data cubes (one per excitation wavelength) projected into the PCs space, whose directions were defined by each eigenvector. Therefore, to achieve dimensionality reduction, certain eigenvectors from the coordinate space were removed according their eigenvalues. Eigenvalues contain variance information about eigenvectors, i.e., the higher the eigenvalue, the more variance that its corresponding eigenvector represents. Therefore, eigenvectors with lower eigenvalues were discarded, as they have less significance.

#### Animals

All experimental procedures were conducted in conformity with European Union Directive 2010/63/EU and were approved by the Ethics Committee for Animal Experimentation of Hospital Gregorio Marañón (Comité de Ética en Experimentación Animal, CEEA; number ES280790000087). All methods were carried out in accordance with relevant guidelines and regulations and in compliance with ARRIVE guidelines. C57BL/6J pregnant mice females (stock 0664, Jackson Labs) were anesthetized by intraperitoneal administration of Ketamine/Xylazine (80 mg/kg and 2 mg/kg, respectively) and then, sacrificed by transcardial perfusion of 20 ml of ice-cold PBS followed by 50 ml of 4% paraformaldehyde (PFA). Embryos were harvested at embryonic day (E) 14.5 and fixed by immersion in 4% paraformaldehyde (PFA) at 4 ^∘^C for 2 h. Afterward, embryos were washed in PBS, and cryopreserved in 30% sucrose. Adult *Neocaridina Davidi* shrimp specimens were handled in compliance with institutional and national animal welfare legislation and maintained according to standard protocols defined by Real Decreto 53/2013. Adult shrimps of 2–6 months old and a size between 2 and 2.5 cm were used in this study.

## Results and discussion

### Single excitation multispectral imaging

As noted in previous section, a whole mouse embryo of 3 cm was analyzed under 405 nm single excitation wavelength. After performing PCA analysis, a total of 62 PCs were found, as there were 62 spectral bands (425–730 nm). The numerical data obtained is summarized in Table 2, showing that PC1 represents the 93.520 % of the total variance, followed by PC2 with a 3.984 %. Although there is a significant disparity in the contribution between these first PCs, the subsequent analysis will demonstrate, through the reconstruction of the RGB image, that these values are indeed sufficient for effectively distinguishing between different tissue types.

**Table 2:** First ten and last PC eigenvalues and variances of mouse whole-body sample.

PC	Eigenvalue	Variance (%)	PC	Eigenvalue	Variance (%)
PC1	1.83e+09	93.520	PC6	1.91e+06	0.098
PC2	7.81e+07	3.984	PC7	1.00e+06	0.051
PC3	3.12e+07	1.592	PC8	7.50e+05	0.038
PC4	7.23e+06	0.369	PC9	6.60e+05	0.034
PC5	3.36e+06	0.185	PC10	3.18e+05	0.016
$\left[ ... \right]$			$\left[ ... \right]$		
PC62	49.95	0.001			

**Figure 6:**
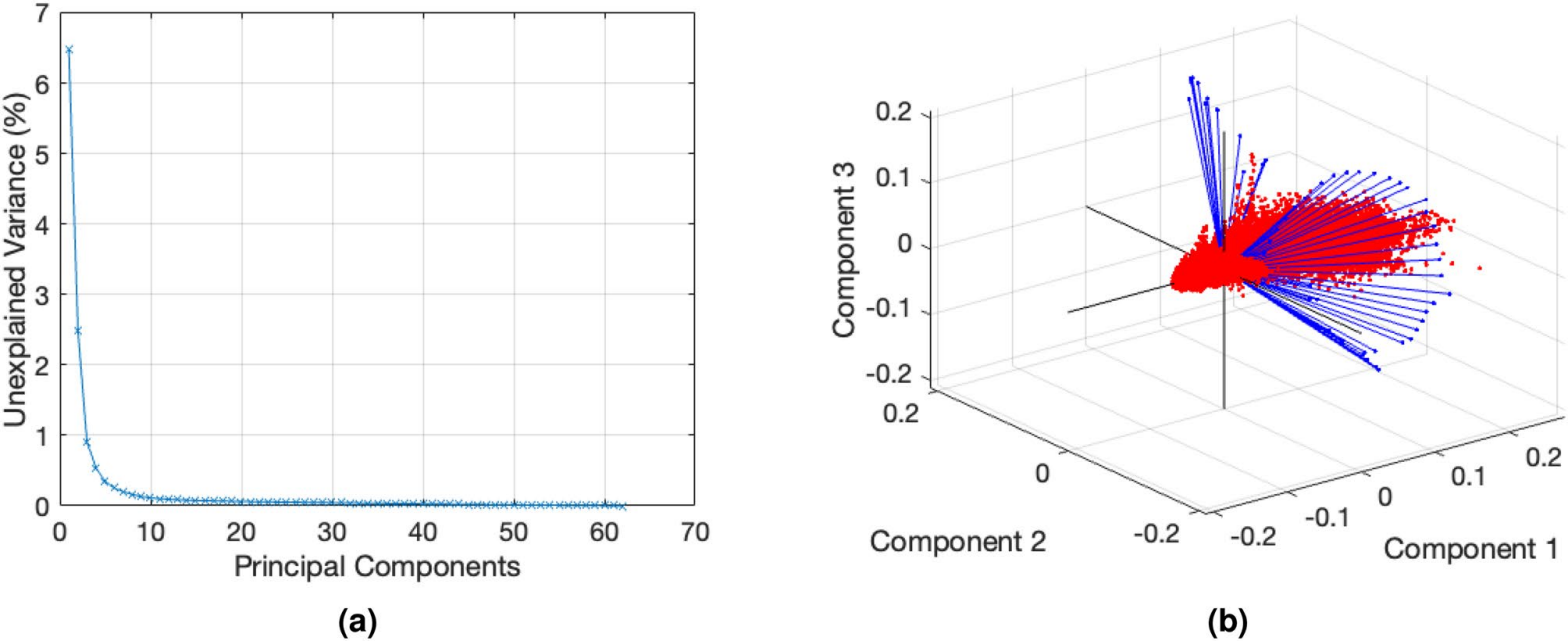
Cumulative sum of the variance of each PC (**a**) and biplot representation of the first three PCs (**b**) of the mouse whole-body sample.

In Figure 6, a dual representation of the 62 PCs acquired is depicted. Figure 6a illustrates the plot of the cumulative sum of the variance (unexplained variance). The first PCs have most of variance representation with a steep slope around the first six PCs. The biplot representation (Fig. 6b) shows the similarity between the first three PCs. The axes corresponds to the coefficients of the PCs, while the blue vectors represent the variables, specifically the 62 different spectral emission wavelengths. The red dots represents the observations, denoting the total number of pixels found in the images. Notably, a significant portion of observations are around the axis defined by the coefficients of the first PCs, signifying a consistent analysis. Furthermore, the uniform distribution of variables (blue vectors) across the space indicates that PC 2 and 3 hold information evenly. This can be also observed in the values presented in Table 2, where variance difference between PC2 and PC3 is approximately 2.39.

**Figure 7:**
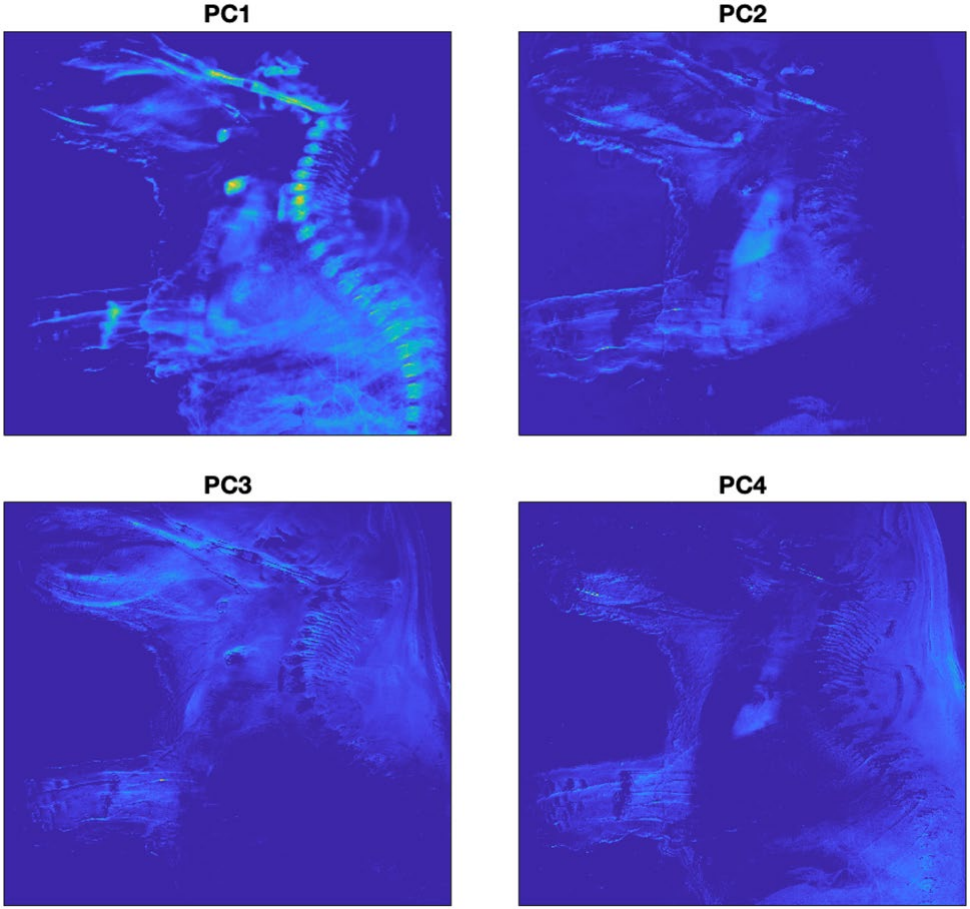
Visualization of four PCs of mouse embryo body.

Figure 7 shows the visualization of the four first PCs in 1024 $\times$ 1024 pixel images. PC1, characterized by the highest variance, extracted a substantial amount of information. Pixels of the entire abdominal part, the spinal cord, the maxilla, bone, and the heart (colored in green color, in front of the upper part of the vertebrae in PC1) are visible. PC2 contains information predominantly from the upper left side, encompassing the arms and the front part of the head. PC3 contains information about the upper back of the mouse, the skull, and the arms. Finally, PC4 reveals peripheral structures, including muscles or skin, as well as leg bone and spine.

For tissue characterization, the first three PCs were used to build the RGB image, as they represented 99.0968% of the total variance. Therefore, reducing the dimensionality of the dataset. PC1 was assigned to the red channel; PC2 to the green channel and PC3 to the blue channel.

**Figure 8:**
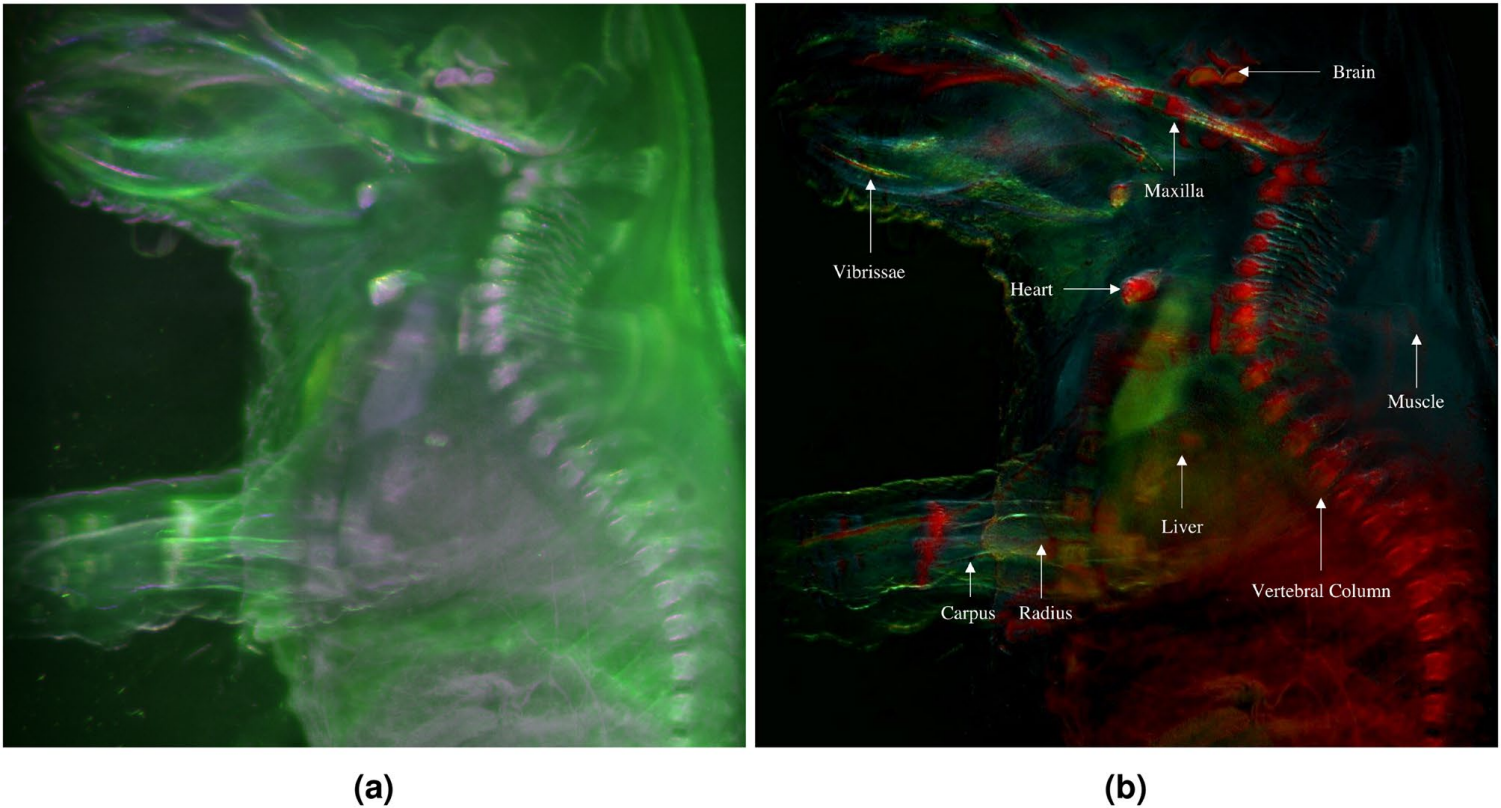
Pseudocolored multispectral image of the mouse body (**a**) and single RGB composite image of the mouse embryo body using PC1, PC2 and PC3 under single excitation multispectral imaging (**b**).

Figure 8 shows the pseudocolored multispectral image of the mouse body without applying PCA, and the final RGB image obtained after merging RGB channels after applying PCA. Tissues like skin and muscle, which appeared indistinguishable and uniformly green in the multispectral image, show varied shades of cyan and green in the PCA image, showing how PCA analysis enhanced tissue differentiation. The heart, brain, abdominal viscera, and major spine bones are predominantly represented in red tones in the PCA image, a distinction also observable in the multispectral image as light pink tones. However, the brain section above the maxilla bone manifests a reddish-orange hue in the PCA image, different to the one exhibited by the other mentioned tissues. Additionally, the central abdominal region also presents a dark orange shade due to liver presence, a differentiation not apparent in the multispectral image, where these tissues are shown in light pink along with bones, brain and heart. Vibrissae are discernible in a combination of green and yellow colors in the PCA image, contrasting with their light pink tone in the multispectral image.

Greater specificity was necessary to discern the subtle variations among the soft tissues in the abdominal area. Therefore, the subsequent analysis aims to enhance the sensitivity of the implemented method, enabling the differentiation of a broader range of tissues.

#### Multi-excitation multispectral Imaging

In the case of multi-excitation multispectral imaging, several lasers were used to excite the sample at different times, as described in Materials section. The four data cubes, acquired with the UV (405 nm), blue (488 nm), green (532 nm), and red (632 nm) lasers, were analyzed. Therefore, a total of 161 PCs were extracted from the analysis. A summary of the eigenvalues and variances of the first ten and last PCs can be found in Table 3. The comparison between these results and those obtained from the single excitation analysis, which considered only the 62 images acquired under UV excitation, shows that the first PC represents a lower percentage of the total variance. Therefore, subsequent PCs carry more significant information. The higher uniformity in the variance distribution allows a finer and more accurate differentiation between the tissues present in the mouse sample. In contrast, the last PC in the multi-excitation modality has a lower variance than that obtained in the single-excitation analysis. However, this does not adversely affect the accuracy of tissue characterization, as only the information from the first PCs was considered in the analysis.

**Table 3:** First ten and last PC eigenvalues and variances of mouse embryo body through multi-excitation multispectral analysis.

PC	Eigenvalue	Variance (%)	PC	Eigenvalue	Variance (%)
PC1	2.40e+09	92.611	PC6	6.28e+06	0.241
PC2	9.28e+07	3.573	PC7	4.18e+06	0.161
PC3	3.35e+07	1.290	PC8	1.92e+06	0.074
PC4	2.75e+07	1.058	PC9	1.36e+06	0.052
PC5	1.66e+07	0.638	PC10	9.87e+05	0.004
$\left[ ... \right]$			$\left[ ... \right]$		
PC161	9.64e+03	3.710e-04			

**Figure 9:**
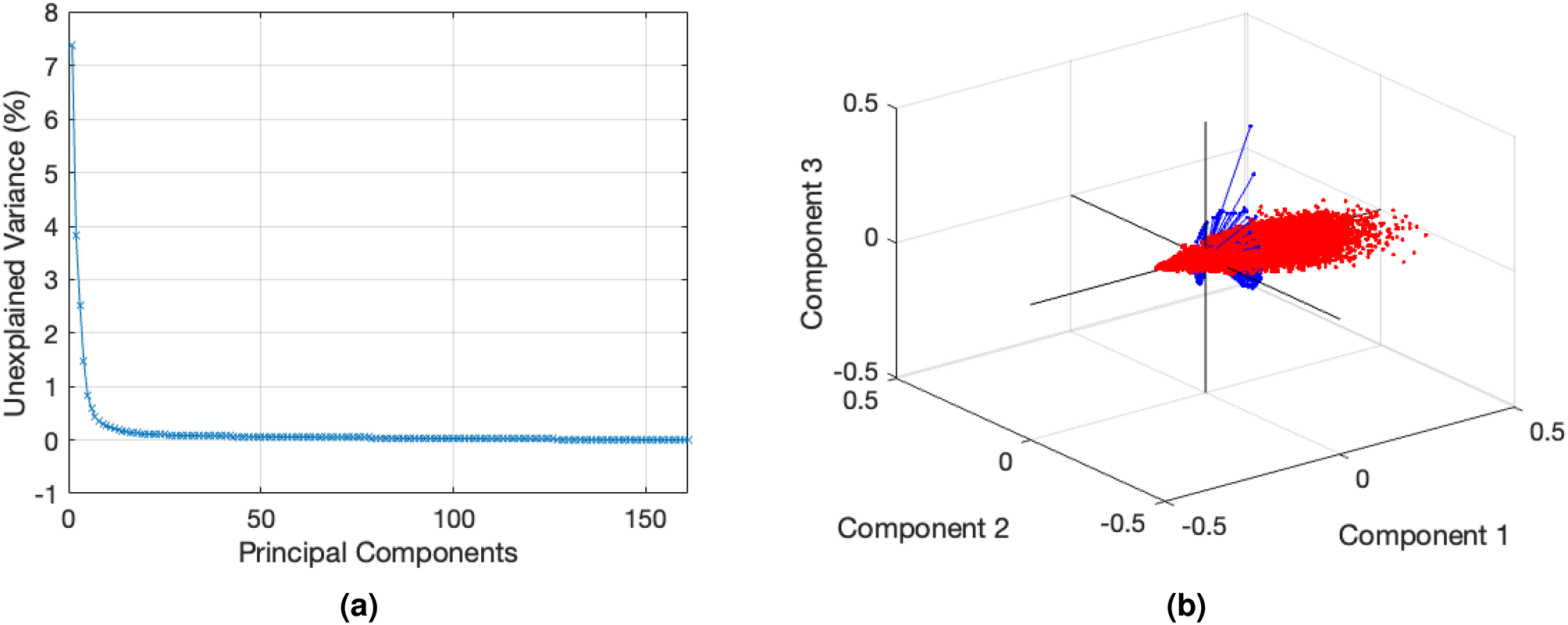
Cumulative sum of the variance of each PC (**a**) and biplot representation of the first three PCs (**b**) of the mouse embryo body through multi-excitation analysis.

The dual representation of the 161 PC acquired for multi-excitation analysis is depicted in Fig. 9. Figure 9a shows that the trend of the unexplained variance for each PC is very similar to that observed in the single-excitation analysis. However, the slope in this case is smother. Since the number of PCs is higher, the unexplained variance is also greater compared to the previous case. Moreover, the cumulative sum of the variances of the first three PCs is equal to 97.474%, whereas in the previous case represented 99.097% of the variance. This lower variance indicates a more uniform distribution of the PCs, thereby resulting in a larger number of PCs with significant information which facilitates tissue separation. In Fig. 9b it can be seen that the observations tends to be distributed along the first PC.

**Figure 10:**
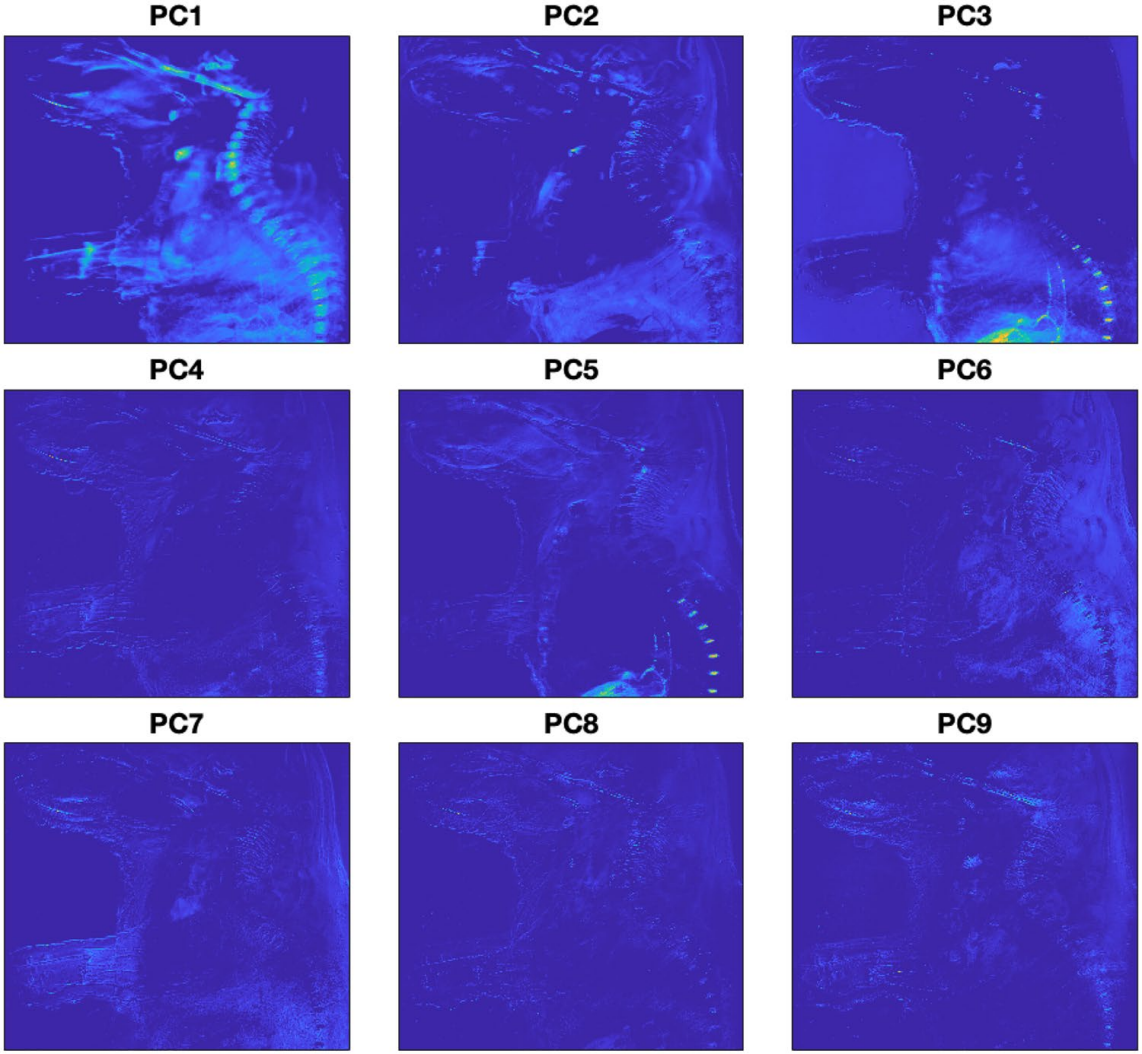
Visualization of the first nine PCs of mouse embryo body through multi-excitation analysis.

Figure 10 shows the visualization of the first nine PCs in 1024 $\times$ 1024 pixel images. The first PC is similar to that obtained through single excitation analysis. However, notable differences are observed in the subsequent PCs. PC2 highlights the lower part of the abdominal cavity and reveals distinct details of the cranial area, such as the maxilla bone and the front of the mouse’s snout. PCs 4, 6, 7, 8, and 9 capture various details of the skin, bones, and muscles. Notably, the intervertebral discs and the large intestine are discernible in the third and fifth PCs. Therefore, this demonstrates how multi-excitation approach enhances the sensitivity of the analysis.

**Figure 11:**
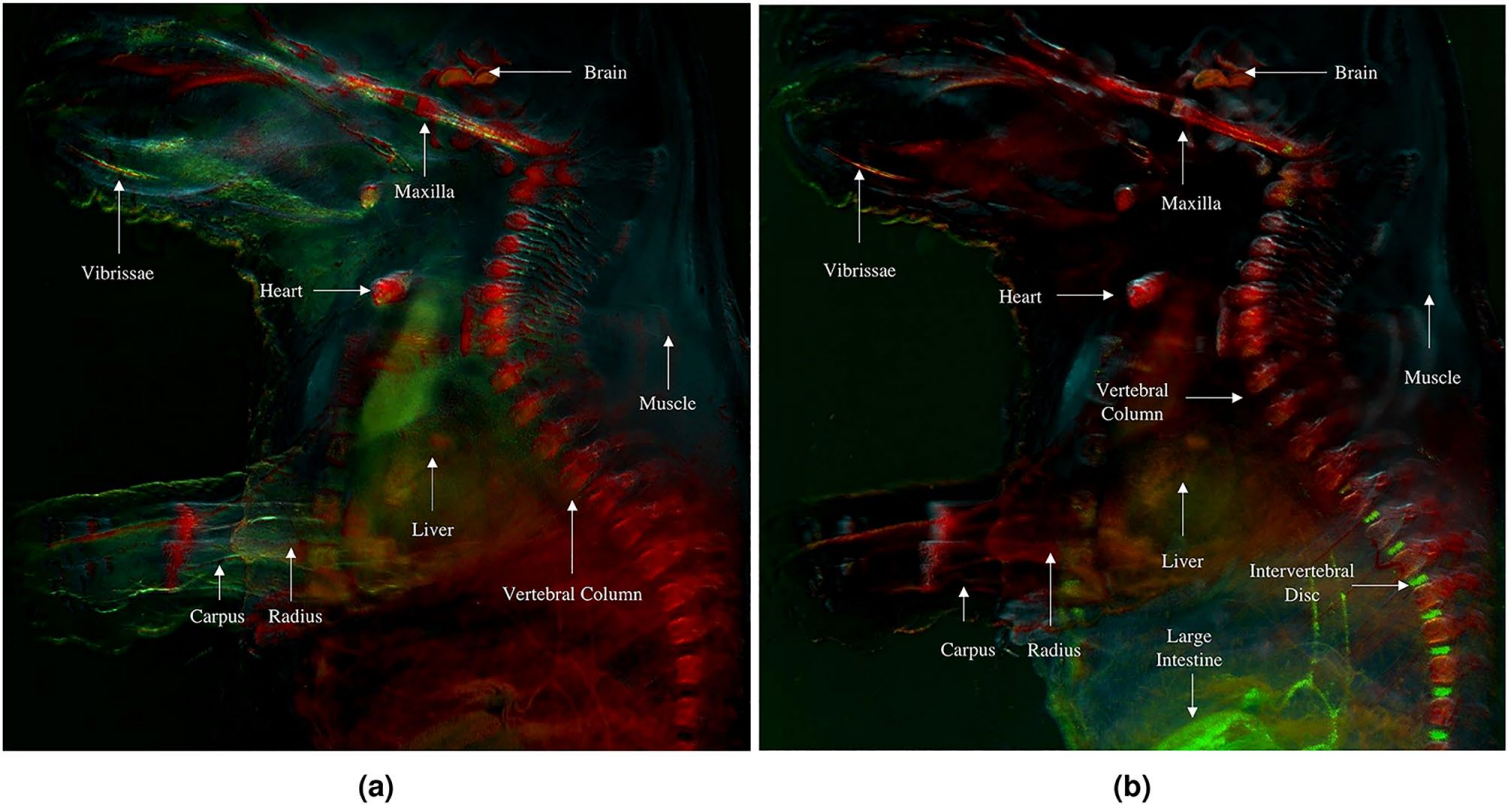
RGB composite image of the mouse whole-body sample using PC1, PC2 and PC3 under single excitation multispectral imaging (**a**) and multi-excitation multispectral imaging (**b**).

For RGB image reconstruction, the first three PCs were used, as they represented 97.4737% of the variability. Similar to the previous case, the assignment of the three PCs to the respective channels of the RGB image was performed. However, in this approach, the PCs were not sequentially introduced into the respective RGB channel. PC3, which contains information about the intervertebral discs and the large intestine as mentioned earlier, was introduced into the green channel to enhance its visibility to the human eye. Figure 11 shows the comparison between the RGB composite image of the mouse embryo body under single excitation multispectral imaging (Fig. 11a) and under multi-excitation multispectral analysis (Fig. 11b) after applying PCA. The introduction of the third principal component (PC) in the green channel enhances the differentiation of soft tissues within the abdominal cavity, including the large intestine, which was not distinguishable in a single-excitation approach. Moreover, blood vessels originating from the large intestine also become discernible. With a multi-excitation approach, clear visualization of the intervertebral discs, located between the vertebrae of the lower spinal column, is also achieved. Additionally, the mouse embryo snout epithelium is detected.

In PC1, bones such as the maxilla, spine, radius, and ulna of the arms are grouped together. The heart and the skin of the lateral part of the mouse embryo muzzle were also grouped in this component. PC2, represented in blue, shows skin and muscle. An orange hue indicates the presence of the brain, liver, and vibrissae. These observations suggest that these tissues possess complex characteristics, and their pixels are formed by the combination of several PCs. Since only three channels were used, it was not possible to assign a specific color to each exclusive tissue. However, through PCA, tissues with similar autofluorescence behavior were grouped, and it is possible to clearly distinguish them in different hues.

#### Single-excitation multispectral Imaging with 3d reconstruction

In this experiment, a living *Neocaridina Davidi* shrimp specimen was excited using a 488 nm blue laser, as mentioned earlier. By capturing the filtered emissions within the 510–730 nm range, with steps of 5 nm, a total of 45 images per optical section in a z-stack were acquired. Each z-stack consisted of 100 optical sections taken from the shrimp for every filtered emission wavelength. Hence, a total of 100 * 45 PCs were generated for the whole volume of the specimen.

As in previous approaches, only the first three PCs from each z-stack were considered, as they exhibited the highest variance and largest eigenvalues. This led to the utilization of 300 PCs for the analysis.

**Figure 12:**
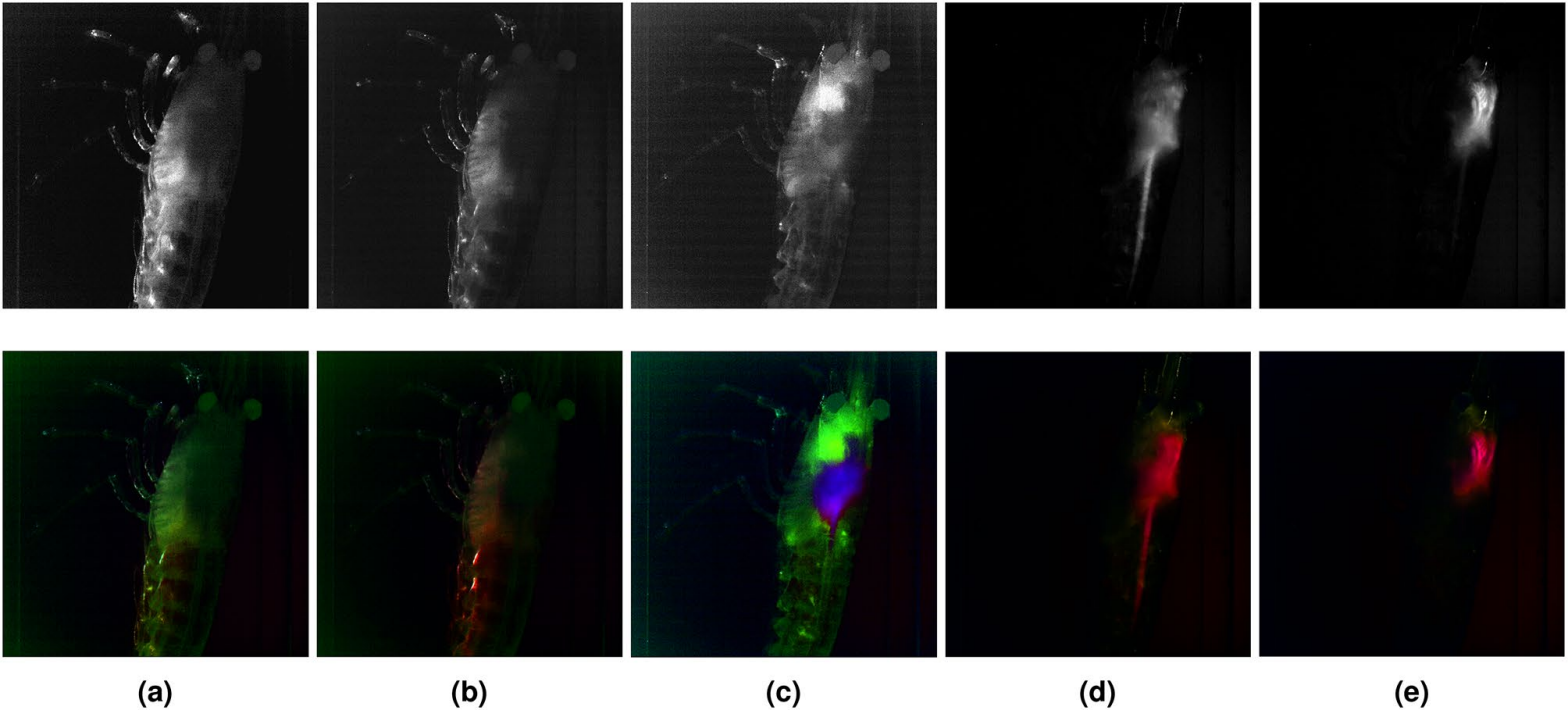
Montage of 5 out of 100 images displaying the intensity composite (upper section) and RGB composite (lower section) created by merging the first three principal components (PCs) of each z-stack of the *Neocaridina davidi* shrimp. Differentiated parts are shown: exoskeleton (**a**), plepodods (**b**), hepatopancreas (**c**), intestine (**d**) and bronchi (**e**).

The groups of 100 images corresponding to each of the three PCs were assigned to an RGB channel and merged together. As the images were merged into three channels, 100 composite images were obtained, i.e. a composite z-stack. Figure 12 shows a representative example of 5 out of 100 intensity and RGB composite optical sections, showing different differentiated parts as exoskeleton (a), plepodods (b), hepatopancreas (c), intestine (d) or bronchi (e). These composite optical sections were used to construct a three-dimensional (3D) volume of the specimen through their projection.

**Figure 13:**
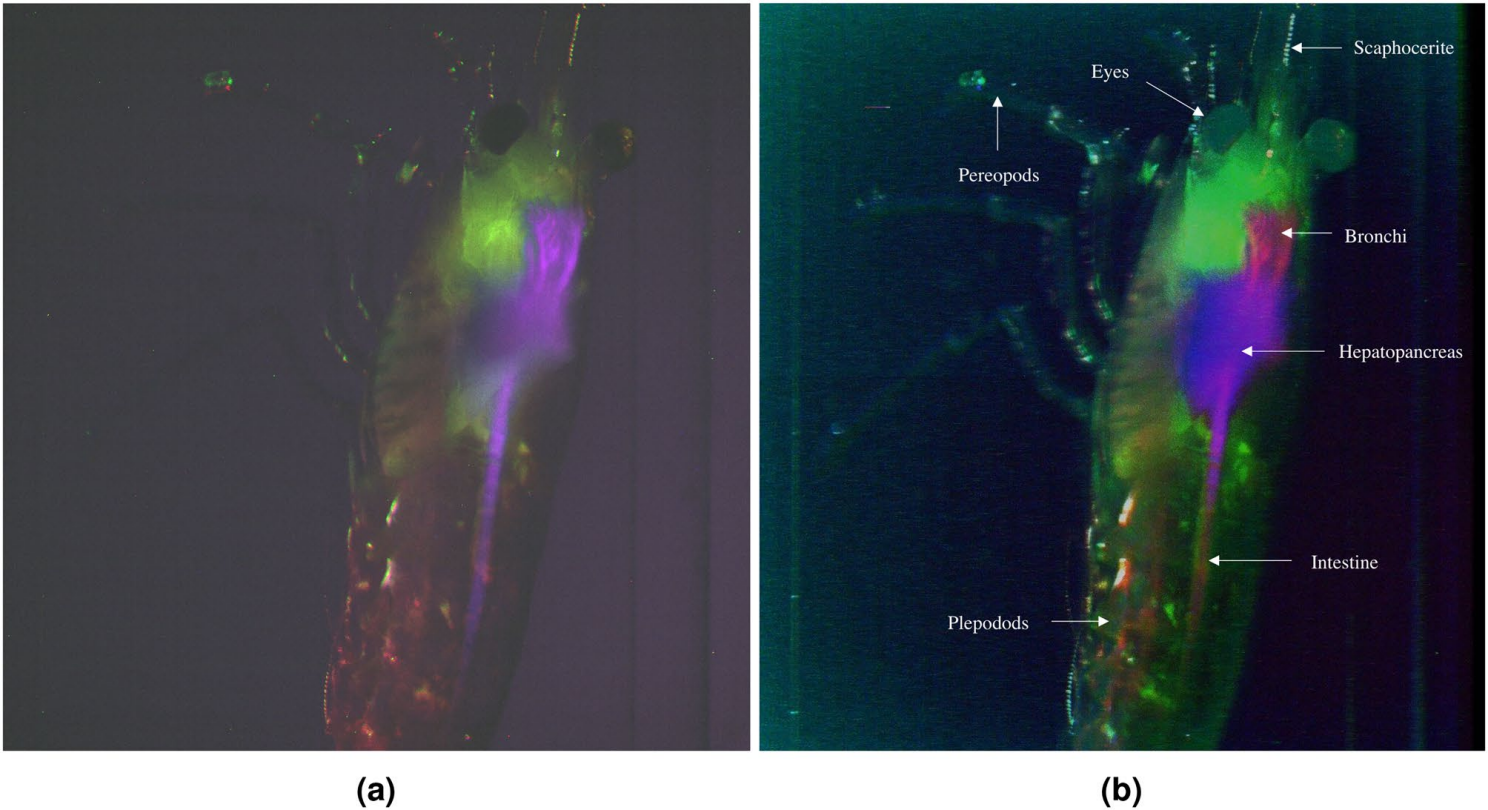
3D projection pseudocolored multispectral image of the specimen without applying PCA (**a**) and 3D projection of the shrimp created from composite optical sections obtained through PCA (**b**).

Figure 13 shows the 2-dimensional representation of the created 3D multispectral pseudocolored volume of *Neocaridina Davidi* shrimp before applying PCA and with labeled parts after applying PCA. The application of PCA allows for the separation of different tissues that were not distinguishable in the pseudocolored multispectral image. The exoskeleton is depicted in green, as this tissue was detected by PC2 and assigned to the green channel. Although the pereopods initially appeared green in the multispectral image, PCA analysis revealed the separation of exoskeleton tissue from the pereopods, which now exhibit a blueish hue due to detection by PC2 and PC3, assigned to the green and blue channels, respectively. Additionally, differentiation is observed between the bronchi, hepatopancreas, and intestine. While these tissues appeared purple in the multispectral image, PCA analysis helps to distinguish them. The hepatopancreas is shown in a purple hue, the intestine in a reddish-green color, and the bronchi in pink, facilitating easier differentiation. On the lateral side of the carapace, the pleopods (shrimp legs) are highlighted in red in the PCA image, differentiated from the exoskeleton. The shape of the pleopods is not fully discernible due to the shrimp’s positioning within the cuvette. However, that part of the shrimp is shown in red colour since the autofluorescence activity of the plepodods differs from the one of the carapace. The scaphocerite is also distinguishable in a blueish-white color.

It is shown how PCA is also able to enhance tissue differentiation in a different, living specimen. In this case, by acquiring information from 100 planes of the shrimp, the amount of information increases and thus increases the sensitivity of the system. This way of proceeding with the acquisition of multispectral images increases the specificity and the number of detected tissues because the light beam sweeps through the sample and excites a greater amount of autofluorescence biomolecules.

## Conclusions

The presented imaging method combines PCA with multispectral imaging, enabling label-free tissue characterization without the need of applying immunohistochemistry (IHC), which is a costly, time-consuming, and expertise-demanding technique.

The initial approach utilizing single excitation analysis successfully differentiated various tissue types in a fixed mouse sample from the first three out of 62 PCs extracted. Therefore, decomposing a complex autofluorescence spectral signal and reducing its dimensionality to only three components. The tissue types differentiated included major spinal bones, the heart, and vibrissae. However, higher specificity was required in order to separate the soft tissues of the abdominal area. Therefore, a multi-excitation analysis method was evaluated. The utilization of multiple excitation lasers allowed for enhanced specificity in tissue characterization, enabling the identification of a broader range of tissues based on their distinct responses to different excitation wavelengths. Furthermore, the developed method was successfully applied to segment tissues in a living sample. By employing a single blue laser for excitation, which ensured the preservation of the living specimen, the entire volume of the sample was acquired and processed using PCA. The amount of analyzed data facilitated the accurate detection of constituent tissues and their subsequent 3D reconstruction. Additionally, the implementation of optical sectioning further improved the ability of PCA to detect different tissues by exciting multiple planes within the sample.

The effectiveness of PCA as a tissue characterization method has been validated for in vitro fixed whole specimens and in vivo whole live specimens. Future research directions should focus on acquiring and assigning emission and excitation spectra for each specific tissue. Obtaining spectral information for each tissue under normal and healthy conditions would enable the distinction of pathological changes for in-vivo studies. Diseased tissues exhibit variations in autofluorescence spectra due to altered metabolism, tissue composition and modified cytoarchitecture. Therefore, the acquisition of standardized spectra would facilitate quick, easy, and precise comparisons of samples under evaluation. This process could involve acquiring images of each tissue to obtain average absorption and emission spectra, and subsequently using PCA to assign PC scores normalized for each tissue based on the inherent characteristics of the sample. Furthermore, this technique could also be extended to the study of isolated organs and tissues. For instance, the heart exhibits a diverse range of textures; the atria differ significantly from the ventricles, as the latter consist of muscular, hollow chambers containing fluid. Similar variations can be observed in the brain, which also features two hollow ventricles. Thus, this approach could be also highly valuable in distinguishing areas of infarction within both the heart and the brain.

## Data Availability

The datasets used and/or analysed during the current study available from the corresponding author on reasonable request.
